# Context dependence in the symbiosis between *Dictyostelium discoideum* and *Paraburkholderia*

**DOI:** 10.1002/evl3.281

**Published:** 2022-05-02

**Authors:** Trey J. Scott, David C. Queller, Joan E. Strassmann

**Affiliations:** ^1^ Department of Biology Washington University in St. Louis St. Louis Missouri 63130

**Keywords:** Bet‐hedging, competition, context dependence, *Dictyostelium discoideum*, *Paraburkholderia*, symbiosis

## Abstract

Symbiotic interactions change with environmental context. Measuring these context‐dependent effects in hosts and symbionts is critical to determining the nature of symbiotic interactions. We investigated context dependence in the symbiosis between social amoeba hosts and their inedible *Paraburkholderia* bacterial symbionts, where the context is the abundance of host food bacteria. *Paraburkholderia* have been shown to harm hosts dispersed to food‐rich environments, but aid hosts dispersed to food‐poor environments by allowing hosts to carry food bacteria. Through measuring symbiont density and host spore production, we show that this food context matters in three other ways. First, it matters for symbionts, who suffer a greater cost from competition with food bacteria in the food‐rich context. Second, it matters for host‐symbiont conflict, changing how symbiont density negatively impacts host spore production. Third, data‐based simulations show that symbiosis often provides a long‐term fitness advantage for hosts after rounds of growth and dispersal in variable food contexts, especially when conditions are harsh with little food. These results show how food context can have many consequences for the *Dictyostelium*‐*Paraburkholderia* symbiosis and that both sides can frequently benefit.

## Impact Summary

Many organisms form symbiotic relationships with other species. These symbioses often exhibit context dependence, where the sign or magnitude of one partner's effect on the other will change in different environments. Context‐dependent effects make it difficult to assign interactions to categories like mutualisms or antagonisms because they involve both benefits and costs depending on the environment. However, in some cases, accounting for context dependence can clarify an interaction so that it more easily fits a mutualism or antagonism. We investigated context dependence using the symbiosis between *Dictyostelium discoideum* and two symbiotic *Paraburkholderia* species. In this symbiosis, *Paraburkholderia* bacteria allow hosts to carry food bacteria to food‐poor contexts, where hosts rarely survive without food, but reduce host fitness in the more hospitable food‐rich contexts. The effect of food context on P*araburkholderia* symbionts is unknown. We show that *Paraburkholderia* symbionts are also affected by this context, through facing reduced competition after being dispersed by hosts to food‐poor contexts. We also identify a new way that symbionts affect hosts, where symbiont density reduces host fitness, but less so in food‐poor contexts. Finally, we use simulations to show that infected hosts benefit in the long term across variable food contexts, especially in the harshest environments with little food. These results show that context dependence in symbiosis can have many consequences for hosts and symbionts, although in general for *D. discoideum* and *Paraburkholderia*, both are likely to benefit.

Context dependence, where the environment can change the sign or magnitude of one partner's effect on the other, is common in symbioses (Bronstein 1994; Thompson 1994; Chamberlain et al. 2014). These context‐dependent effects on partners can be crucial to understanding the nature of symbiotic interactions (Keeling and McCutcheon 2017; Iwai 2019). For example, in the symbioses between *Paramecium bursaria* hosts and their *Chlorella* endosymbionts, hosts benefitted from symbiosis in light environments, but were harmed in the dark. For *Chlorella*, the effects of symbiosis were negative in co‐culture, indicating that hosts exploited their endosymbionts for the benefits hosts receive in light conditions (Lowe et al. 2016). However, in the context of an environment with a *Chlorella* competitor, hosts benefited their symbionts by eating these competitors (Iwai 2019). This example illustrates that understanding how partners affect each other across multiple contexts can change our view of the interaction, sometimes from one of exploitation to one of mutual benefit.

Context dependence is important in the lifecycle of the social amoeba *Dictyostelium discoideum*. Amoebae need edible bacteria to grow and proliferate (Raper 1937), but the abundance (Young 2004; Vos et al. 2013) and quality (Kuserk 1980; Brock et al. 2018) of food bacteria in the soil is known to vary. This results in a patchy environment where some patches are food rich and other patches are food poor. In response to starvation, amoebae aggregate and form a multicellular fruiting body to disperse resistant spores to new environments (smith et al. 2014). The patchy soil environment is considered an important selection pressure for this fruiting body structure (Bonner 1982; Kessin 2001).

*Dictyostelium discoideum* interacts with three species of mostly inedible *Paraburkholderia* bacterial symbionts—*P. agricolaris*, *P. hayleyella*, and *P. bonniae* (Brock et al. 2020). Throughout this article, we will use “*Paraburkholderia*” or “symbionts” as shorthand for the three symbiotic *Paraburkholderia* species. Hosts infected with *Paraburkholderia* have been isolated from multiple locations in the United States, with around 25% of screened hosts being infected by at least one species (Haselkorn et al. 2019). *Paraburkholderia* are able to enter and live inside *D. discoideum* cells and spores, but can also proliferate, albeit sometimes only slowly, without their hosts (DiSalvo et al. 2015; Shu et al. 2018a; Brock et al. 2020) unlike the obligate endosymbionts that are also found in *D. discoideum* (Haselkorn et al. 2021). There is some evidence consistent with coevolution between hosts and symbionts (Brock et al. 2016; Shu et al. 2018a; Garcia et al. 2019; Brock et al. 2020). For example, host clones naturally infected with *P. hayleyella* are harmed less by infection with this symbiont than host clones that were not infected in the wild (Shu et al. 2018a) indicating that *P. hayleyella* hosts have adaptations favoring symbiosis. Symbionts also have the ability to move toward hosts (Shu et al. 2018b), suggesting that being able to find hosts is beneficial.

The symbiosis with *Paraburkholderia* bacteria impacts the growth and proliferation of *D. discoideum*. Having symbionts allows hosts to carry food bacteria (and inedible *Paraburkholderia*) inside the spore‐containing part of fruiting bodies called the sorus (DiSalvo et al. 2015). Whether this novel trait is advantageous or not depends on the presence of food bacteria after dispersal. When food is abundant, having symbionts can be costly, as shown by infected amoebae producing fewer spores than uninfected amoebae (Brock et al. 2011; DiSalvo et al. 2015). In food‐poor environments, the cost of having *Paraburkholderia* is compensated by hosts gaining the ability to carry food bacteria in dispersing spores. This allows amoebae to disperse and grow where they ordinarily could not (Brock et al. 2011; DiSalvo et al. 2015). These context‐dependent effects on the host could be extremely important in the natural soil environment, where food‐poor patches arise frequently (Kessin 2001).

Less is known about how symbionts are affected across food contexts. Gaining the ability to disperse to new locations may be a major reason for symbionts to seek out social amoeba hosts (Garcia and Gerardo 2014), but could also make the context of host food bacteria in the new environment important for symbionts. A benefit from being dispersed to patches with few bacteria could be that symbionts face reduced competition. If few bacteria are present, symbionts will mostly compete with food bacteria that were also carried in the sorus. This should be a relatively low competition situation because symbionts outnumber food bacteria in sori (Khojandi et al. 2019). Having few competitors should advantage symbionts, whereas environments with plentiful bacteria could strongly limit symbiont growth because of their relatively slow growth rates, at least as measured in the lab (Brock et al. 2020). We will use “food‐rich” and “food‐poor” to describe newly colonized patches with many and few bacteria, respectively, of the sort edible by *D. discoideum*. These categories reflect the relationship to *D. discoideum* and could be called high and low competition in terms of their effect for *Paraburkholderia*.

It is unclear how the number of extracellular *Paraburkholderia* in the environment impacts hosts because previous studies have focused on intracellular *Paraburkholderia* (Shu et al. 2018b; Miller et al., 2020). When they are outside the amoebae, *Paraburkholderia* could affect *D. discoideum* fitness through interactions with food bacteria perhaps by reducing the amount of food for hosts through competition or by releasing diffusible toxins that affect amoebae. Thus, host food context could also affect the relationship between symbiont density and host spore production.

The fitness effects of symbiosis for hosts have been tested only in food‐poor and food‐rich contexts individually. The benefits of symbiosis could pay out over the long term across different food contexts in the soil. Growth rates in temporally variable contexts are best captured by geometric mean fitness rather than arithmetic mean fitness because only the geometric mean captures the lasting effects of periods of low fitness (Sæther and Engen 2015). Ignoring geometric means can lead to incorrect assessments of the adaptive value of strategies in variable environments. One example of an adaptation that is only apparent from geometric mean fitness measures is bet‐hedging phenotypes, where organisms adapt to uncertain environments by avoiding the worst effects of harsh contexts while being suboptimal in more favorable contexts (Slatkin 1974; Philippi and Seger 1989; Starrfelt and Kokko 2012). This lowers the variance in fitness across time and results in higher geometric mean fitness at the expense of lower arithmetic mean fitness.

Bet‐hedging is suspected to play a role in explaining observations of disadvantageous partnerships in plant‐fungus mutualisms (Lekberg and Koide 2014; Veresoglou et al. 2021). If bet‐hedging occurs, short‐term costs are acceptable if partnerships increase geometric mean fitness. Alternatively, symbiosis could increase both geometric and arithmetic mean fitness across contexts without the need for bet‐hedging. In this case, the benefits of symbiosis simply outweigh the costs as in more traditional descriptions of symbioses (Douglas 2010). However, these alternatives have not been tested in detail.

To understand context dependence in the symbiosis between amoebae and *Paraburkholderia*, we used *D. discoideum* infected with either *P. agricolaris* or *P. hayleyella*—the two most common and best‐studied species of *D. discoideum* symbionts. We investigate whether *Paraburkholderia* benefit from reduced interspecific competition when dispersed to food‐poor contexts, how symbiont density and food context impact host spore production, and whether symbiosis is beneficial for hosts when food conditions vary.

## Methods

**Figure 1:**
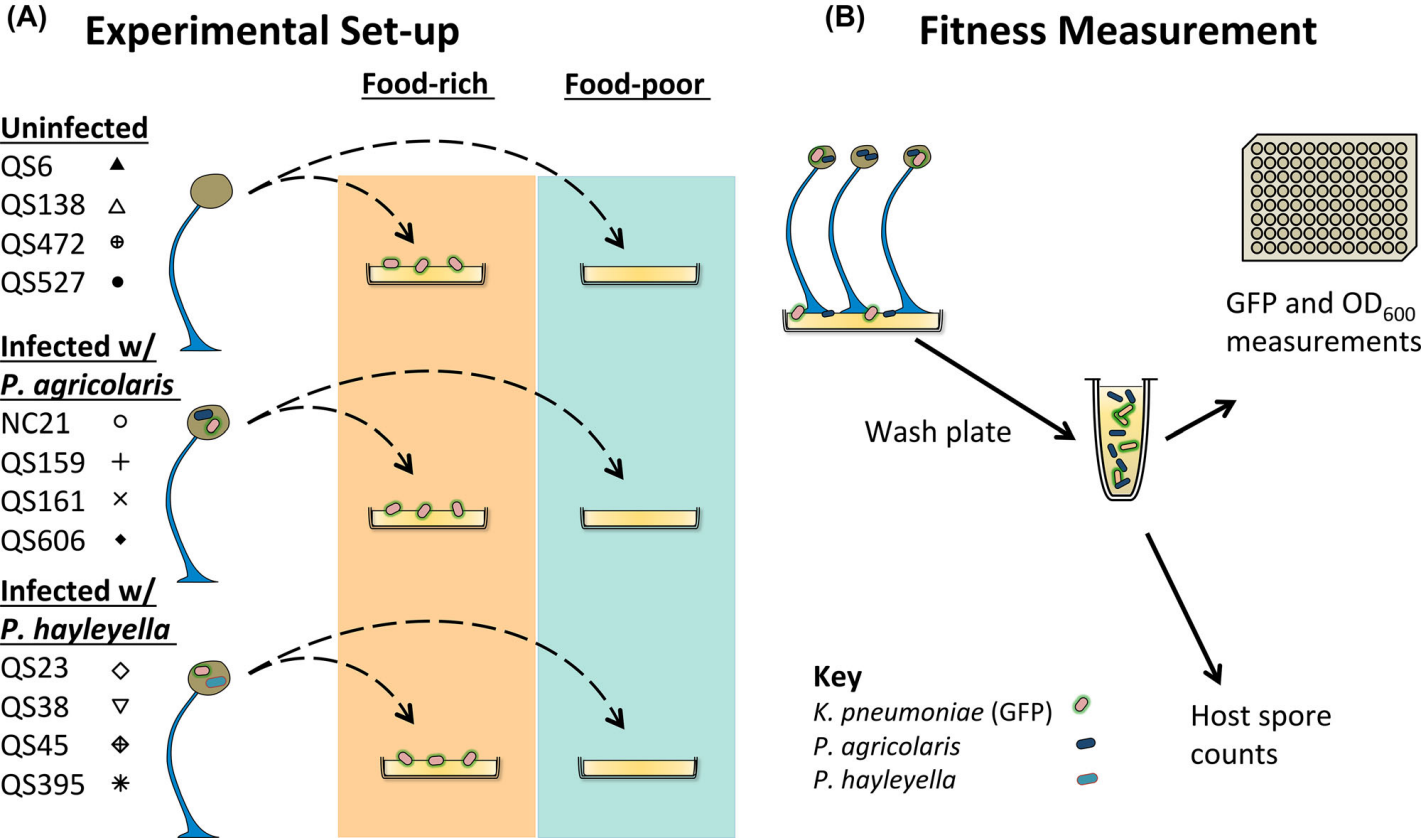
Schematic of experimental design. (A) Uninfected and infected *D. discoideum* fruiting bodies are collected and plated on food‐rich and food‐poor plates (after one passage on GFP‐expressing *K. pneumoniae* food bacteria). These plates are grown for 6 days and then washed for bacterial measurement and spore counting (B). Bacteria are measured by calculating GFP fluorescence and optical density (see *Methods*). Host spore production is measured from washed plates.

To understand the effects of symbiosis across food contexts, we used four naturally uninfected *D. discoideum* clones, four clones naturally infected with *P. agricolaris*, and four clones naturally infected with *P. hayleyella* (Fig. 1). We cured the infected clones and re‐infected them with their native symbionts to standardize infection density. Uninfected clones were left uninfected, but were otherwise treated the same as infected clones. This resulted in three host infection conditions: uninfected, infected with *P. agricolaris*, and infected with *P. hayleyella*. To mimic natural dispersal, we collected sori and transferred them to food‐rich (with additional *K. pneumoniae* bacteria) or food‐poor (KK2 buffer with no *K. pneumoniae*) nutrient plates. Bacteria appear on food‐poor plates only if transferred sori contain bacteria, as expected for infected samples. We grew replicate experimental sets involving all conditions beginning on two separate dates, July 13 and 22, 2020, and followed up with additional experiments (see *Results*) beginning on January 26 and April 23, 2021.

### *Paraburkholderia* ISOLATION

To isolate *Paraburkholderia* from their hosts, we grew wild collected *D. discoideum* clones on SM/5 plates (2 g glucose [Fisher Scientific], 2 g Bacto Peptone [Oxoid], 2 g yeast extract [Oxoid], 0.2 g MgSO_4_ * 7H2O [Fisher Scientific], 1.9 g KH_2_PO_4_ [Sigma‐Aldrich], 1 g K_2_HPO_4_ [Fisher Scientific], and 15 g agar [Fisher Scientific] per liter). Wild *D. discoideum* clones were grown with *K. pneumoniae* food bacteria that were suspended in KK2 buffer (2.25 g KH_2_PO_4_ [Sigma‐Aldrich] and 0.67 g K_2_HPO_4_ [Fisher Scientific] per liter). After wild clones completed the social cycle (feeding, starvation, and fruiting body formation), we collected sori with pipette tips and placed them on SM/5 plates. We allowed the bacteria and amoebae contained within to proliferate and then streaked out the resulting bacteria to get single colonies.

### *Paraburkholderia* REMOVAL

To generate uninfected clones, we treated infected *D. discoideum* clones with antibiotics by plating on 30 μg/mL tetracycline SM/5 plates with 200 μL of 1.5 optical density (OD_600_) tetracycline‐resistant *K. pneumoniae* suspended in KK2 buffer. After passage on SM/5 plates without tetracycline to let the amoebae recover from any effects of the antibiotic, we collected single sori with a pipette tip and placed 10 of them in different locations on SM/5 plates to confirm that we had successfully removed the bacteria. If bacteria are present, these spot tests will show bacterial growth and *Dictyostelium* proliferation as the spores hatch and eat the bacteria (Brock et al. 2011). Without bacteria, amoebae cannot proliferate and the spot will stay blank. We considered a clone to be cured if no bacteria showed up in spot tests. We similarly treated naturally uninfected hosts with tetracycline to control for any effect of curing on our results.

### *Paraburkholderia RE‐* INFECTION

We re‐infected cured *D. discoideum* clones with their native *Paraburkholderia* isolates by plating 200 μL 2 × 10^5^ spores with 200 μL of 0.1% *Paraburkholderia* solution. This solution consisted of 1.5 OD_600_ *Paraburkholderia* and 1.5 OD_600_ *K. pneumoniae* in a 1:1000 ratio. To confirm re‐infection (and also successful isolation), we performed spot tests as above, where successful re‐infection was inferred when bacteria grew on eight or more spots out of the 10 we put down.

### ARTIFICIAL DISPERSAL TO FOOD‐RICH AND FOOD‐POOR PLATES

To obtain sori to transfer to food‐rich and food‐poor plates, we started by growing *D. discoideum* clones from frozen stock, as described above, on 200 μL 1.5 OD *K. pneumoniae* expressing green fluorescent protein (GFP). We obtained GFP‐expressing *K. pneumoniae* (strain ID DBS0349837) from the Dicty Stock Center at dictyBase (Fey et al. 2013). This initial growth period is to remove freezer effects and ensure that food bacteria that are carried to new plates are GFP expressing because stocks were fed non‐GFP bacteria before freezing. After 6 days of growth, we used pipette tips to collect sori from mature fruiting bodies. We counted spores using a hemocytometer and diluted spores to a concentration of 2 × 10^5^ per mL, and then plated them on plates with (food‐rich) or without (food‐poor) an additional 200 μL of the GFP‐expressing food bacterium *K. pneumoniae* (Fig. 1). To survive on food‐poor plates, the host must carry food bacteria from the previous plate. We grew food‐rich and food‐poor plates for 6 days unless otherwise stated, enough time for mature fruiting bodies to form.

### MEASUREMENT OF BACTERIA DENSITY

To measure *Paraburkholderia* density, we measured the quantity of bacteria left on plates after *D. discoideum* formed fruiting bodies. We first collected plate contents by washing plates with 15 mL of KK2 buffer. To remove fruiting bodies and bacteria associated with fruiting bodies, we centrifuged wash solutions for 3 minutes at 13,000 rpm. We measured bacteria using optical density measured at 600 nm (OD_600_), a frequency at which bacteria commonly scatter light. Because the OD_600_ is due to both *Paraburkholderia* and *K. pneumoniae*, we used GFP fluorescence measurements (with an excitation wavelength of 485 and emission wavelength of 515 nm) and a standard curve relating *K. pneumoniae* fluorescence to its OD_600_ to subtract out the component due to GFP‐expressing *K. pneumoniae*. Both OD_600_ and fluorescence measures were performed in a 96 well plate with a Tecan Infinite 200 Pro microplate reader.

To validate our standard curve, we compared predicted OD_600_ of *P. agricolaris* and *K. pneumoniae* to colony forming unit (CFU) counts from the same samples. Linear regression revealed that predicted OD_600_ measurements explained most of the variation in CFUs, showing that our assay is reliable (Fig. S1). We also checked our standard curve for significant quadratic terms, which can cause measurement errors when combining OD_600_ and fluorescence measures at high densities (Meyers et al. 2018), but our curve did not have a significant quadratic term.

### HOST SPORE PRODUCTION

Spore production is a standard fitness measure in *D. discoideum* (Buttery et al. 2009; Hall et al. 2013; Gruenheit et al. 2017). To measure host spore production, we estimated spore concentration in the supernatants from washed plates using a hemocytometer. We then calculated the total number of spores per plate by multiplying by the volume of wash solution.

### SPORE PRODUCTION SIMULATIONS

To test whether infected hosts benefit across variable food contexts, we simulated rounds of growth and dispersal across soil patches with different probabilities of having food bacteria. We separately modeled three host phenotypes: (1) uninfected, (2) infected with *P. agricolaris*, and (3) infected with *P. hayleyella*. Co‐infections are possible, but are rare in nature (Haselkorn et al. 2019), so we exclude them from our analysis.

We assumed that environments consisted of 100 discrete soil patches. Patches were either food poor or food rich (we investigated continuous amounts of food and found similar results; see File S1). Food‐poor patches at time *t* were drawn from a binomial distribution with probability *p_t_*. Food‐rich patches were drawn with probability 1 – p*_t_*. To allow temporal variation, the value of *p_t_* in each generation was drawn from a beta distribution with mean *p* and variance *v_t_*_emp_. High values of *v*_temp_ resulted in more temporally variable environments. For low values, most of the variation was spatial.

Initially all patches were colonized. Each patch produced a number of spores, drawn from the distribution of our empirical spore production values, according to whether it was a food‐rich or food‐poor patch. To model costs, we penalized host spore production in food‐rich environments by reducing spore production by a percentage *c*. When *c* is 0, we modeled the scenario observed in this study, with no infection cost. We did not detect a cost of infection in food‐rich contexts, but numerous other studies have documented this cost (Brock et al. 2011; DiSalvo et al. 2015; Miller et al., 2020). It is likely that we did not detect a cost because we infected hosts with fewer *Paraburkholderia*. Because these costs have been demonstrated repeatedly in other studies and because of the importance of costs to bet‐hedging (Lekberg and Koide 2014; Veresoglou et al. 2021), we included them as a variable. We summed cost‐adjusted spore production values to get the total spore production across all patches. This is divided by 2 × 10^5^, a rough estimate of the number of spores in a typical sorus, to get the total number of sori, which we are assuming to be the dispersal unit.

The global pool of sori is used to seed the next round. New patches are assumed to be empty and dispersal is assumed to be global such that sori from one patch can disperse to any other patch with equal probability. Dispersal is likely efficient in *D. discoideum* as sori can be dispersed long distances by arthropods (smith et al. 2014) and possibly even by birds (Suthers 1985). Each sorus is randomly assigned to a patch and it successfully colonizes that patch *g*% of the time. Because the value of *g* for natural hosts is unknown, we investigated three values of *g* (50%, 5%, and 0.5%) that range from cases where there are many more sori successfully establishing than available patches to cases where each patch produces around one sorus. We assumed that patches colonized by multiple sori were the same as singly colonized patches for the purposes of determining their subsequent spore production. Some patches may remain unfilled (although this is unlikely when *g* = 50%). We also assume that infection status is not associated with different rates of colonization as those differences are better captured by our empirical spore production values, which will include differences in growth efficiency or spore germination rate.

We vary the average probability of food‐poor patches *p* from 0.1 to 0.9 and simulate four different cost regimes reflecting variation found in different *Paraburkholderia* isolates (Miller et al., 2020). We simulated dispersal to new patches for 100 rounds of growth and dispersal using 100 replicates for each combination of, *v*_temp_, and *c* for each phenotype. Within each replicate, all three phenotypes experience the same environment. At the end of the 100 rounds, we calculated the total spore production per round and calculated geometric and arithmetic mean spore production from these values across the 100 rounds. Within each replicate, we determined whether infected hosts had higher geometric or arithmetic mean fitness for each individual simulation and whether any phenotype went extinct.

We assigned outcomes for each parameter combination by calculating the frequency that infected or uninfected hosts had higher geometric mean fitness or arithmetic mean fitness. Infected and uninfected hosts were assigned as winners if they had higher geometric mean fitness in 75% of replicates. We assigned an outcome as bet‐hedging when infected hosts won and more than half of the winning replicates did so with lower arithmetic mean fitness. Extinctions occurred in some simulations and were treated as a distinct outcome. We assigned mixed outcomes when neither infected nor uninfected hosts were able to have higher geometric mean fitness in 75% of replicates. Some mixed outcomes involved individual replicates where infected hosts were found to bet‐hedge.

### STATISTICAL METHODS

We performed statistics in R version 3.6.3 (R Core Team 2020). To compare bacteria density and spore production, we used linear mixed models (LMM) with the *lme* function in the nlme package (Pinheiro and Bates 2006). To account for random variation from replicate clones and effects of dates when experiments were performed, we included clone and the date the experiment was performed—along with each variable on its own—as random effects. To select the best model of random effects, we used AICc, a sample‐size‐corrected measure of model fit that balances predictive ability and model complexity (Burnham and Anderson 2004). Many of our models showed different variances between treatments. To account for these differences in variance, we weighted models with the *varIdent* function in nlme (Pinheiro and Bates 2006). We used the emmeans package (Lenth et al. 2018) to perform contrasts.

To understand how *Paraburkholderia* density affects host spore production across food conditions, we fit an LMM using only infected hosts that included symbiont density leftover on plates and whether the plate was food rich or food poor, along with the interaction between these variables. We included random effects for clone, date, and both crossed effects and selected the best random effect structure with AICc. We determined whether the interaction was important by comparing AICc of the model including the interaction with models including the other variables but lacking the interaction.

## Results

### *Paraburkholderia* DISPERSED BY *Dictyostelium* SORI HAVE LOWER GROWTH WHEN HOST FOOD BACTERIA ARE ABUNDANT

**Figure 2:**
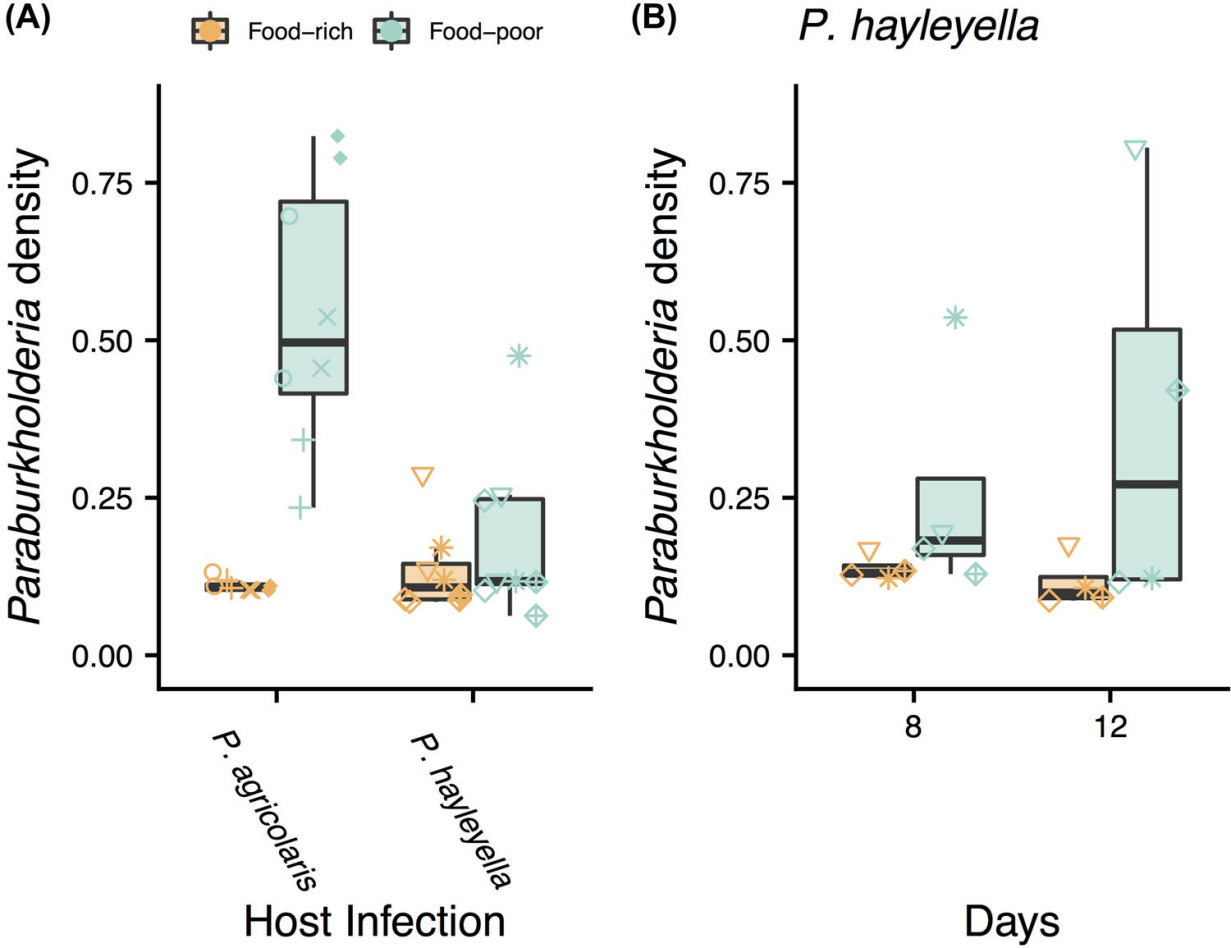
More *Paraburkholderia* were recovered from plates after fruiting body formation from food‐poor plates (those that had not received additional *K. pneumoniae*). (A) *Paraburkholderia* density after 6 days. (B) *Paraburkholderia hayleyella* density after 8 and 12 days. Point shapes show individual clones (see Fig. 1).

The context of a food‐poor environment is known to be important for *D. discoideum* hosts. It is not known how *Paraburkholderia* are affected by this same context, but reduced competition with food bacteria seems likely. We tested this by growing infected sorus contents on food‐poor and food‐rich nutrient plates and measuring the density of *Paraburkholderia* after *D. discoideum* fruiting body formation (Fig. 1). After infected hosts formed fruiting bodies, *Paraburkholderia* densities were lowest in food‐rich conditions (Fig. 2A), as expected if they compete with food bacteria. There was around five times more *P. agricolaris* on food‐poor than food‐rich plates (LMM, *P* < 0.001). *Paraburkholderia hayleyella* growth was higher in food‐poor conditions than food‐rich, but this difference was not significant after 6 days (LMM, *P* = 0.416). Because *P. hayleyella* grows slowly, we performed two more experiments with *P. hayleyella* with 8‐ and 12‐day growth periods (Fig. 2B). Allowing for longer incubations did not result in significantly higher density of *P. hayleyella* (LMM, *P* = 0.633), suggesting that *P. hayleyella* reach their maximum density at or before 6 days, but including these additional experiments gave us enough power to find a significant increase in *P. hayleyella* density in food‐poor conditions relative to food‐rich (LMM, *P* = 0.027). These results show that symbiont density is context dependent.

### HIGHER SYMBIONT DENSITY HARMS HOSTS, BUT LESS SO IN FOOD‐POOR CONTEXTS

**Figure 3:**
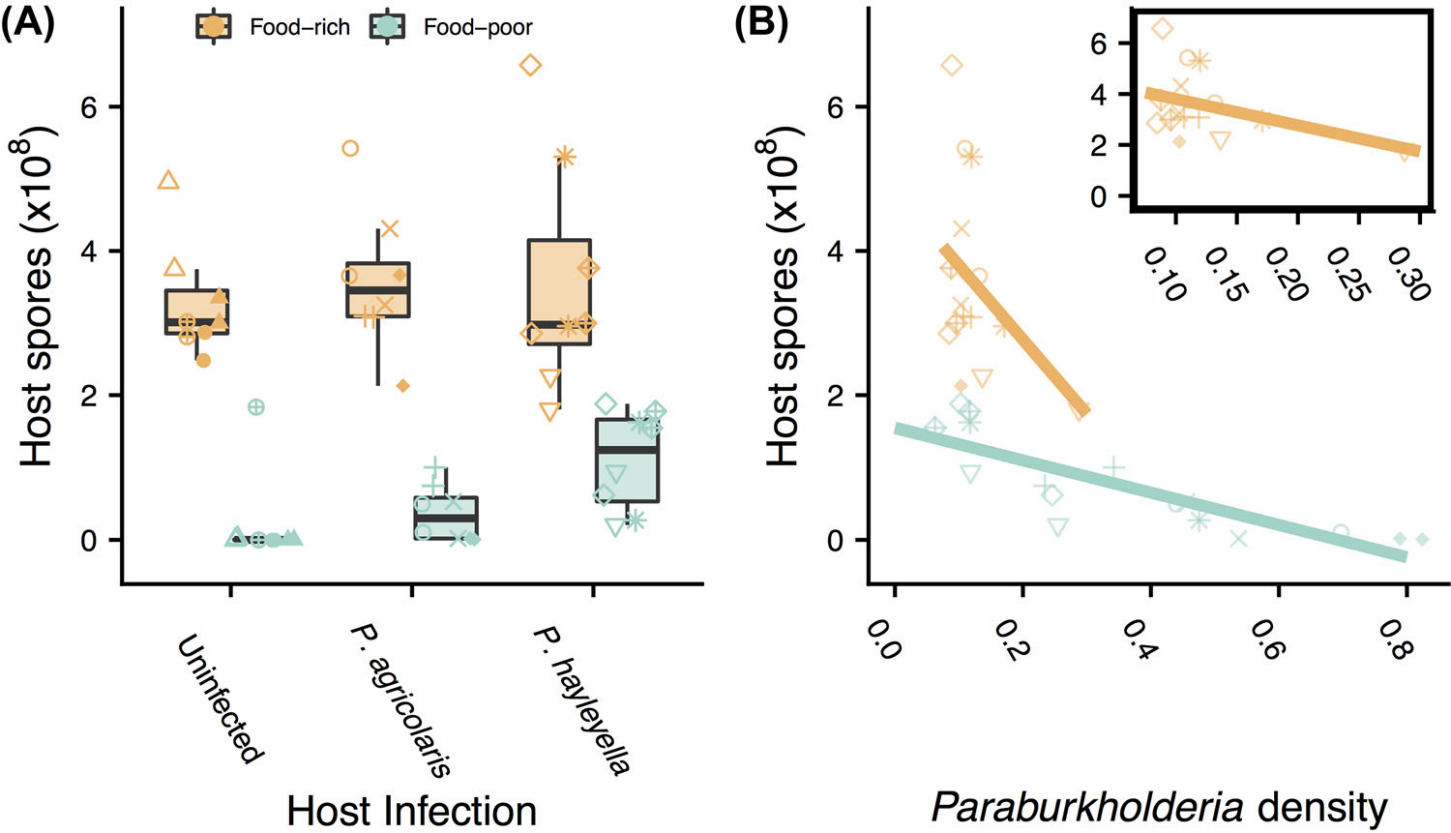
Effects of *Paraburkholderia* infection and density on host spore production. (A) Spore production of hosts from food‐rich and food‐poor plates for uninfected, *P. agricolaris*‐infected, and *P. hayleyella*‐infected hosts. (B) Interaction between measured *Paraburkholderia* density (OD_600_) and food environment on host spore production. This interaction model explained 95% of the variance in spore production. Inset shows food‐rich results on smaller scale. Point shapes show individual clones (see Fig. 1).

The host food context may affect the relationship between symbiont density and host spore production and therefore the degree of conflict or cooperation between them. To investigate this, we also measured total host spore production from plates where we measured the growth of *Paraburkholderia* symbionts (Fig. 1). We used uninfected hosts as a baseline for fitness without symbionts. We confirmed prior studies (Brock et al. 2011; DiSalvo et al. 2015) showing that infected hosts could carry food bacteria and proliferate on food‐poor plates, whereas uninfected host could not (Fig. 3A). Surprisingly, we did not observe a cost of being infected in food‐rich conditions (*P* > 0.5 for both species) which has been seen in previous studies (Brock et al. 2011; DiSalvo et al. 2015; Shu et al. 2018a). This is likely a result of our lower infection dosage of 0.1%.

Although having some symbionts is essential for hosts to be able to carry food and survive in food‐poor conditions, higher symbiont densities may nevertheless harm hosts, perhaps in ways that depend on food context. We found that larger populations of symbionts as measured by OD_600_ were associated with lower host spore production, but this harm was reduced in food‐poor conditions. Lower host spore production was associated with being in a food‐poor environment (β_food‐poor_ = −3.283, SE = 0.853) and symbiont density (β_density_ = −10.317, SE = 6.364), but the interaction between food scarcity and symbiont density showed that the harmful effect of higher symbiont densities was lessened on food‐poor plates (β_food‐poor*density_ = 8.078, SE = 6.381; Fig. 3B). These results indicate that symbiont density may come at the expense of host spore production, but that this cost decreases in food‐poor environments.

### SYMBIOSIS IS OFTEN BENEFICIAL FOR HOSTS ACROSS VARIABLE CONTEXTS

Because symbiosis helps hosts in food‐poor contexts, we hypothesized that infected hosts would gain a long‐term benefit across contexts compared to uninfected hosts. If infected hosts increased their geometric mean fitness at the expense of arithmetic mean fitness, infected hosts could even gain a bet‐hedging advantage. We modeled this by using our empirical spore production values to simulate 100 rounds of growth and dispersal across environments where the number of food‐poor patches was determined by the mean frequency (*p*) and the temporal variance (*v*_temp_; more detail can be found in *Methods*). Because the natural conditions of this symbiosis are mostly unknown, we simulate a wide range of parameter space to determine which conditions favor symbiosis. The supplement includes animations of representative simulations.

**Figure 4:**
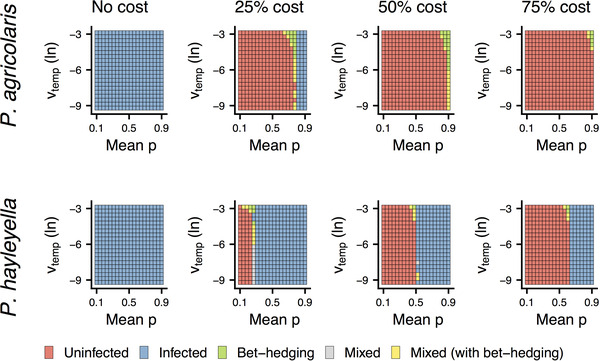
Benefits of symbiosis depends on variation in food availability and fitness costs. Winning phenotypes of *P. agricolaris* (top) and *P. hayleyella* (bottom) relative to uninfected for different costs of infection with a 5% probability of colonization. Orange shows when uninfected hosts have higher arithmetic and geometric mean spore production; blue shows when infected hosts have higher arithmetic and geometric mean spore production; green shows when arithmetic fitness is reduced for higher geometric mean fitness (bet‐hedging); gray shows areas where both infection strategies can win; yellow shows where both strategies can win and where infected hosts bet‐hedge.

We first describe the results when dispersing sori successfully colonize new patches 5% of the time. When there was no cost of infection, we found that infected hosts were favored in every condition we tested (Fig. 4, blue). We also simulated costs of infection because those have been found in other studies (Brock et al. 2011; DiSalvo et al. 2015; Miller et al., 2020). As the cost of infection in food‐rich contexts increased, infected hosts were favored in the most food‐poor environments, whereas uninfected hosts were favored when food was abundant (Fig. 4, orange). *Paraburkholderia hayleyella* was favored across more environments than *P. agricolaris*.

Bet‐hedging in this symbiosis appears to be rare (Fig. 4, green and yellow). Infected hosts had a bet‐hedging advantage when costs were added and food was intermediately rare. More temporally variable environments had a weak effect on increasing the likelihood of bet‐hedging.

When dispersing sori successfully colonize new patches 50% of the time (each patch produces enough sori to completely fill the patches in the next generation), we found similar results (Fig. S2). When only 0.5% were successful (each patch may only produce one or two sori for dispersal), we again found similar results except in the most food‐poor conditions, where both uninfected and *P. agricolaris*‐infected hosts tended to go extinct (Fig. S3A). *Paraburkholderia hayleyella*‐infected hosts were able to survive in these food‐poor contexts (Fig. S3B).

The natural environment of hosts is unlikely to involve food patches that are binary. Variation in the environment is also often autocorrelated, with the state of the environment at one time more often resembling the state of the environment in the near future (Ruokolainen et al. 2009). To determine whether our results were robust to variable environments with continuous food and temporal correlations, we ran additional simulations (described in detail in File S1) where the amount of food varied from 0 to 1 (Fig. S4) depending on a continuous resource that allowed us to tune autocorrelations (Fig. S5). These additional simulations broadly supported our conclusions from the simpler simulations (Fig. S6–S8).

## Discussion

Our results show how the context of host food abundance affects the *Dictyostelium*‐*Paraburkholderia* symbiosis beyond the previously demonstrated advantage to hosts when food is rare (Brock et al. 2011). First, we found evidence that both *Paraburkholderia* species benefit from reduced competition when they are carried to food‐poor environments (Fig. 2). Second, symbiont density negatively affected host spore production, but symbionts harmed hosts less in food‐poor conditions (Fig. 3B). Third, infected hosts had an advantage over uninfected hosts in simulations when food conditions were harsh or when the cost of symbiosis was low (Fig. 4).

Our finding that symbionts had higher growth when dispersed to food‐poor contexts shows that *Paraburkholderia* symbionts experience parallel context dependence as hosts. These results highlight the importance of context dependence for both partners. *Paraburkholderia* may benefit from reduced competition when hosts bring them to food‐poor environments because symbionts interact with fewer competitors or because hosts eat competitors. This, together with our finding that hosts can benefit across contexts, points to a relationship of mutual benefit in this symbiosis. Our results also fit with other findings of competitive benefits for symbionts (Iwai 2019). Other benefits of symbiosis for *Paraburkholderia* remain to be tested.

Competition between symbionts and food bacteria may also be responsible for the context‐dependent effects of symbiont density on host spore production. Our spore production results showed that higher symbiont densities resulted in lower host spore production, indicating that symbionts are harmful to hosts. However, higher symbiont densities are less harmful in food‐poor conditions when competition is lower (Fig. 3B). The reduced harm for hosts could be the result of less antagonism between bacteria, which results in less collateral damage to amoebae through secreted toxins or other competitive interactions between food bacteria and symbionts. The generality of our results is limited somewhat by only using one species of food bacteria. Although using a single food bacterium is more experimentally tractable, amoebae encounter multiple bacteria species in their natural environments (Brock et al. 2018). Different species, or combinations of species, could change competition with symbionts and affect host spore production in different ways.

Symbiosis benefits amoeba hosts by giving hosts the ability to carry food to food‐poor contexts (Brock et al. 2011; DiSalvo et al. 2015). Using simulations, we showed that this ability resulted in higher fitness across variable contexts when costs were low and food was rare (Fig. 4). Under conditions with plentiful food and high costs, being uninfected was advantageous. In nature, about 25% of clones are infected (Haselkorn et al. 2019), suggesting that symbiosis is not universally favored. This indicates that our finding of no cost to hosts in the symbiosis may be unrepresentative of many natural infections. On the other hand, a 25% infection rate is high if the symbiosis is generally harmful. This indicates that the prevalence of symbiosis could reflect a balance of forces where *D. discoideum* is not strongly selected to fight *Paraburkholderia* infection in a geographic mosaic of coevolution (Thompson 1994). Unfortunately, the natural conditions of this symbiosis are the biggest unknowns in this system as it is difficult to study this symbiosis, and microbes more generally (Kraemer and Boynton 2017), in nature.

Hosts could also benefit across contexts through bet‐hedging, where geometric mean fitness trades off with arithmetic mean fitness (Seger and Brockmann 1987). It is suspected that costly symbioses may be able to evolve because they are advantageous over the long term even if they are not advantageous in the short term (Lekberg and Koide 2014; Veresoglou et al. 2021). We found that bet‐hedging was rare in our simulations. Our finding that bet‐hedging occurs between where conditions favor infected over uninfected hosts hints at the possibility that bet‐hedging could facilitate the evolution of symbiosis where benign environments transition to harsh environments. However, as our simulations also reveal, symbiosis is more often favored without the need for bet‐hedging even with costs. Our results thus weaken the case that costly symbiosis in some contexts involves bet‐hedging because symbiosis was more often favored outright than by bet‐hedging.

Symbiotic interactions may play a larger role in adaptation to variable environments than previously understood, even without bet‐hedging. Symbioses are known to result in novel phenotypes that allow partners to survive in harsh conditions (Moran 2007; Oliver et al. 2010). Rarely do studies incorporate environmental variation and long‐term fitness. We investigated the long‐term effects of context dependence in the symbiosis between *D. discoideum* and *Paraburkholderia* and found that hosts frequently benefited from symbiosis in the harshest conditions. An understanding of the ecological contexts along with long‐term measures of fitness will be important for understanding the evolutionary consequences of context‐dependent symbioses.

## AUTHOR CONTRIBUTIONS

TS, DQ, and JS designed the study and wrote the manuscript. TS performed the experiments and simulations and analyzed the data.

## DATA ARCHIVING

Data and code for simulations and analysis are available at www.gitlab.com/treyjscott/farmerBH and are archived on Dryad (https://doi.org/10.5061/dryad.2ngf1vhqp).

## CONFLICT OF INTEREST

The authors declare no conflict of interest.

Associate Editor: A. Gardner

## Supporting information


**Figure S1**: Linear regression results relating estimated optical density (predicted from standard curve) to colony counts from serial dilutions of the same *P. agricolaris* (A) and *K. pneumoniae* (B) samples
**Figure S2**: Plot of the best phenotype for a given probability of being in a no food environment and cost of having symbionts.
**Figure S3**: Plot of the best phenotype for a given probability of being in a no food environment and cost of having symbionts
**Figure S4**: Models of continuous food without and with costs. Points show empirical spore production values from Figure 3
**Figure S5**: Temporally and spatially varying resource and its impact on food.
**Figure S6**: Plots of best phenotypes from continuous food simulations.
**Figure S7**: Plots of best phenotypes from continuous food simulations.
**Figure S8**: Plots of best phenotypes from continuous food simulations.

SUPPORTING MATERIAL
