# Supplementary figures and images for: Context dependence in the symbiosis between Dictyostelium discoideum and Paraburkholderia
Source: Evol Lett. 2022 May 2;6(3):245–54. doi: 10.1002/evl3.281 (PMC9233174; doi:10.1002/evl3.281)

## Slide 1
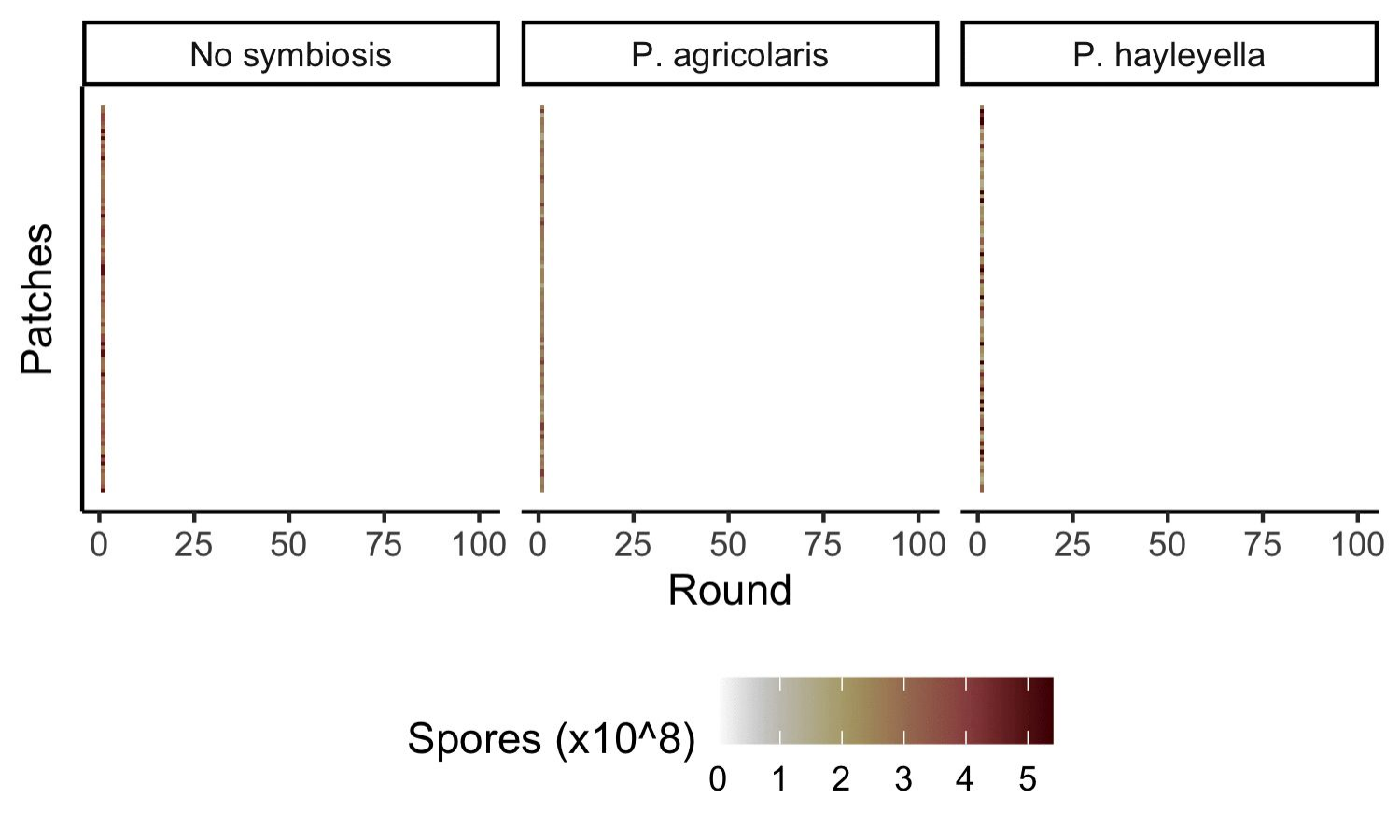

## Slide 2
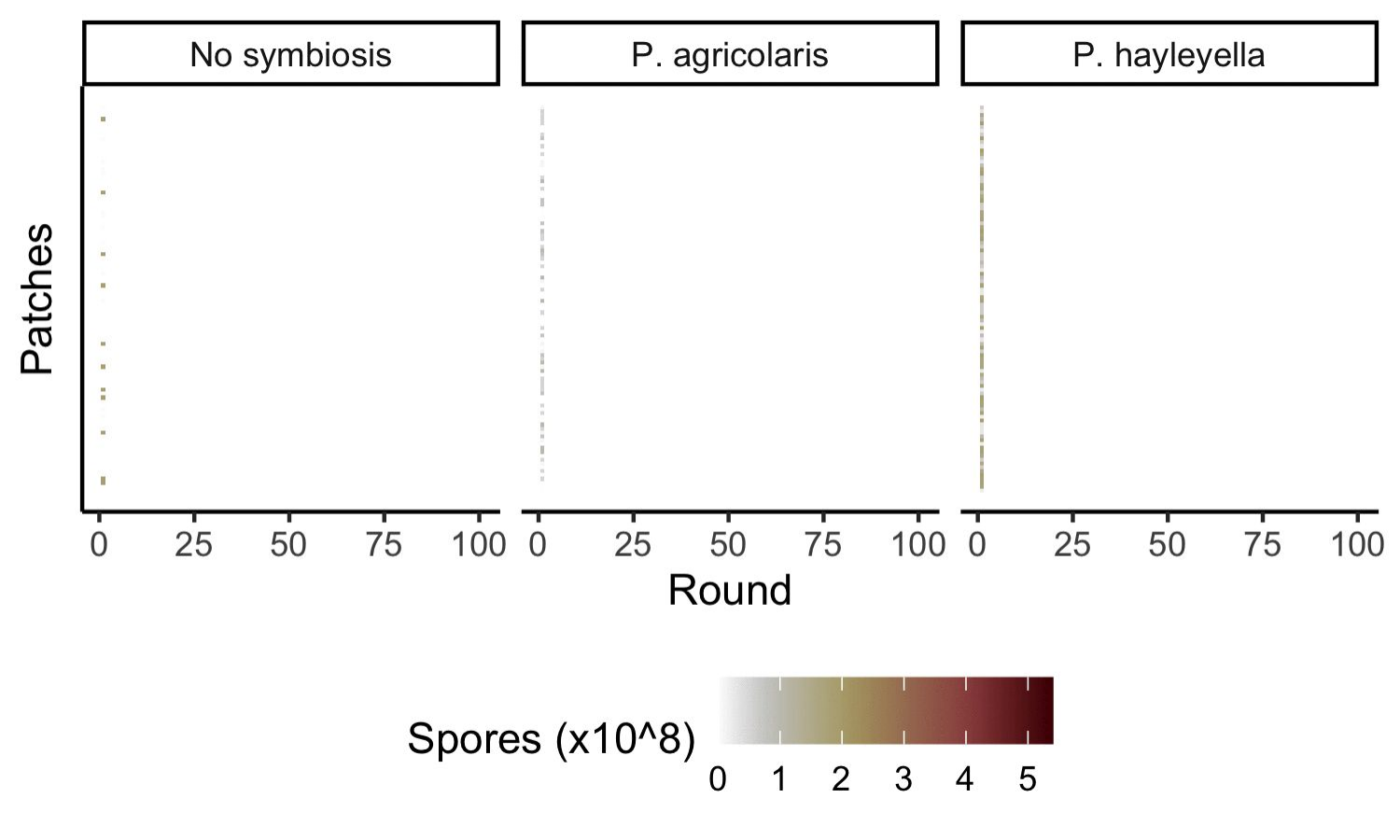

## Slide 3
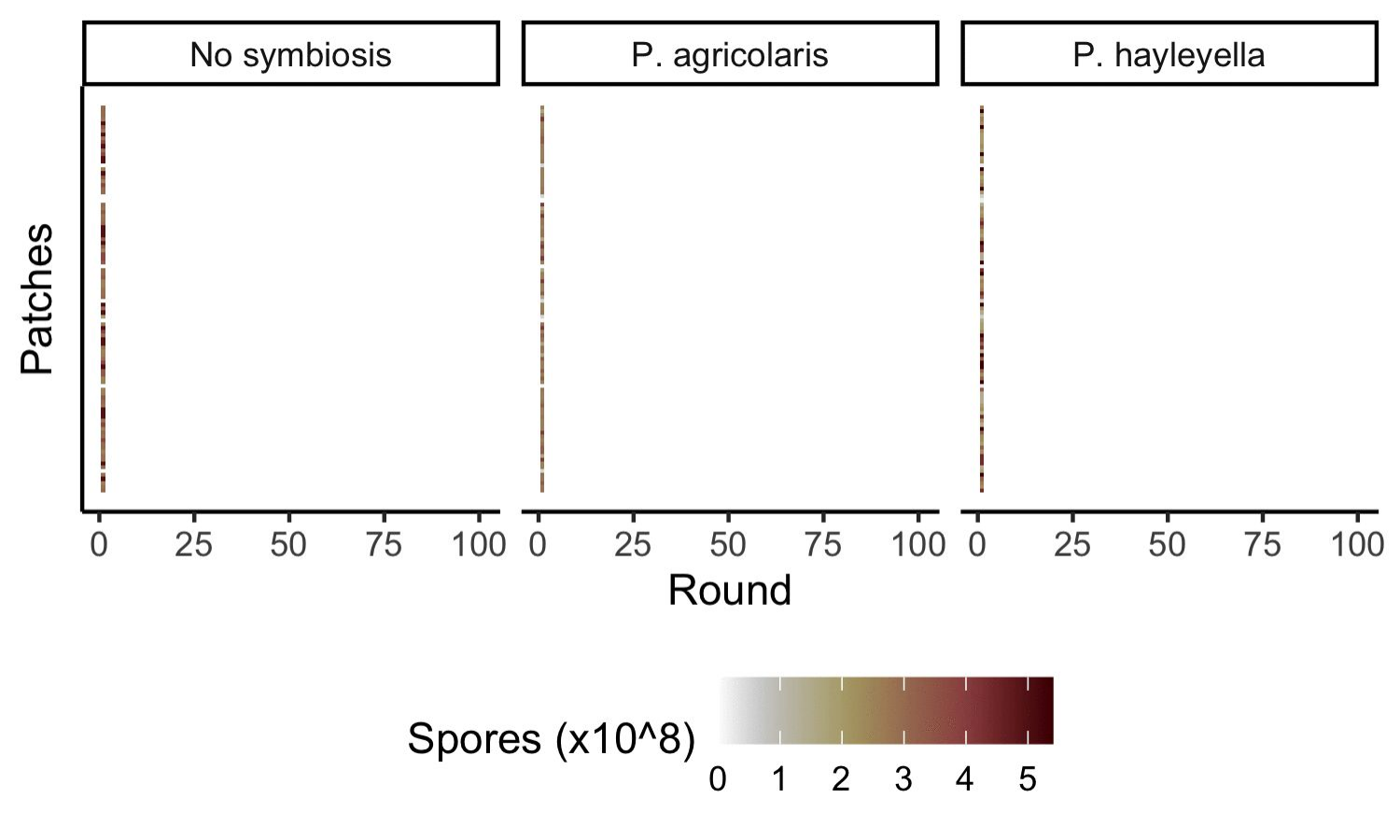

## Slide 4
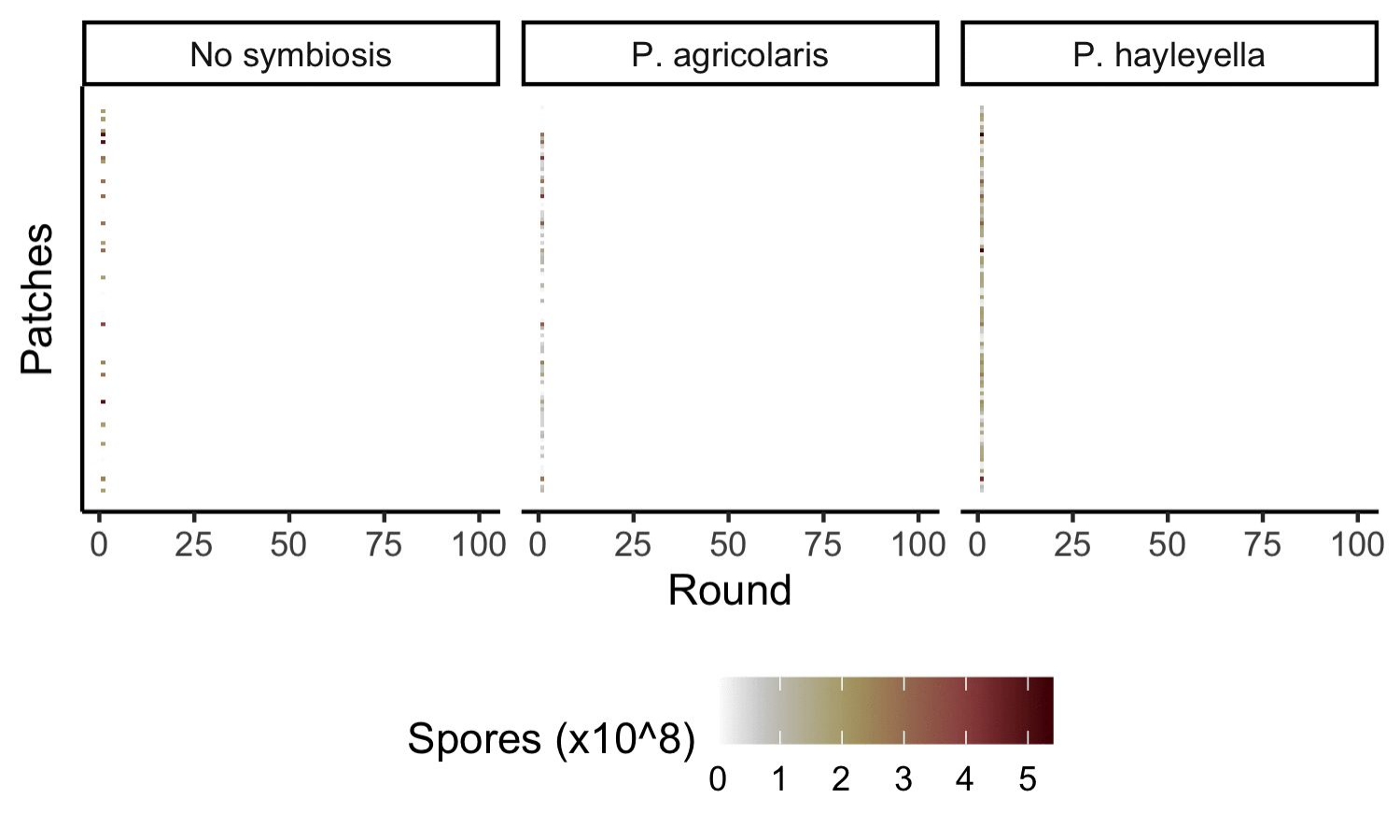

Supplement: Supplementary file 2 — SUPPORTING MATERIAL [file EVL3-6-245-s002.pptx]
